# Changes in PCSK 9 and apolipoprotein B100 in Niemann–Pick disease after enzyme replacement therapy with olipudase alfa

**DOI:** 10.1186/s13023-021-01739-y

**Published:** 2021-02-27

**Authors:** Bethanie Garside, Jan Hoong Ho, See Kwok, Yifen Liu, Shaishav Dhage, Rachelle Donn, Zohaib Iqbal, Simon A. Jones, Handrean Soran

**Affiliations:** 1 Lipid Research Group, Division of Cardiovascular Sciences, University of Manchester, Manchester, UK; 2 Cardiovascular Trials Unit, Manchester Royal Infirmary, Manchester University NHS Foundation Trust, Oxford Road, Manchester, M13 9WL UK; 3 Faculty of Biology, Medicine and Health, University of Manchester, Manchester, UK; 4 Manchester Centre for Genomic Medicine, St Marys Hospital, Manchester University NHS Foundation Trust, Manchester, UK

**Keywords:** Acid sphingomyelinase deficiency, Apolipoprotein B100, Proprotein convertase subtilisin/klexin type 9, Tumour necrosis factor α, Low density lipoprotein cholesterol, Enzyme replacement therapy

## Abstract

**Background:**

Enzyme replacement therapy (ERT) with olipudase alfa, a recombinant human acid sphingomyelinase (rhASM), is being developed to treat patients with ASM deficiency (ASMD), commonly known as Niemann–Pick disease (NPD) types A or B. This study assessed the effect of ERT on lipid parameters and inflammatory markers.

**Methods:**

Serum and plasma samples from five adults with NPD type B (NPD-B) who received olipudase alfa ERT for 26 weeks were analysed. We also collected fasting blood samples from fifteen age- and sex-matched participants as reference and comparison group. We measured fasting lipid profile, apolipoproteins B48 and B100 (apoB48 and apoB100), apolipoprotein A1 (apoA1), proprotein convertase subtilisin/klexin type 9 **(**PCSK9) mass, oxidised low-density lipoprotein (oxLDL), small dense low-density lipoprotein cholesterol (sdLDL-C) and tumour necrosis factor α (TNF-α).

**Results:**

Patients with NPD-B, compared with age and sex matched reference group, had higher triglycerides, PCSK9, apoB48, oxLDL and TNF-α and lower high density lipoprotein cholesterol (HDL-C) and apoA1. Treatment with ERT was associated with improved lipid parameters including total cholesterol, triglycerides, low density lipoprotein cholesterol (LDL-C), sdLDL-C, oxLDL and apoB100. Though there was an increase in apoA1, HDL-C was slightly reduced. TNF-α showed a reduction. ApoB100 decreased in parallel with a decrease in total serum PCSK9 mass after ERT.

**Conclusion:**

This study demonstrated that patients with NPD-B had a proatherogenic lipid profile and higher circulating TNF-α compared to reference group. There was an improvement in dyslipidaemia after olipudase alfa. It was possible that reductions in LDL-C and apoB100 were driven by reductions in TNF-α and PCSK9 following ERT.

## Background

Acid sphingomyelinase deficiency (ASMD), also known as Niemann–Pick disease (NPD) types A or B, is an extremely rare genetic disorder characterised by mutations in the SMPD1 (sphingomyelin phosphodiesterase 1) gene, leading to a deficiency of the enzyme acid sphingomyelinase (ASM). NPD type C is not considered in this study as it has a different causality. ASMD leads to an accumulation of sphingomyelin and large lipid-laden foam cells within the hepatocytes as well as tissues within the lung, spleen, lymph nodes, adrenal cortex, bone marrow and central nervous system [1–3].

NPD type A (NPD-A) is a rapidly progressive fatal neurodegenerative disorder leading to death by 2 to 3 years of age. NPD type B (NPD-B) has little or no neurological involvement and most patients survive into adulthood. NPD Type B is characterised by hepatosplenomegaly, thrombocytopaenia, interstitial lung disease and dyslipidaemia [1–3]. Lipid profiles of NPD patients are characterised by elevated LDL-C, very low-density lipoprotein (VLDL) cholesterol, and triglyceride levels, whereas HDL-C levels are significantly reduced [4]. This atherogenic lipid profile has contributed to the onset of early coronary artery disease in NPD-B patients [1, 3].

While there is currently no approved therapy for NPD, enzyme replacement therapy (ERT) with recombinant acid sphingomyelinase (olipudase alfa) is being developed as a treatment option. The atherogenic lipid profiles in NPD-B patients were shown to improve with ERT in a phase 1b clinical trial (DFI13412 study, NCT01722526) [4]. Frozen serum and plasma samples of the patients collected as part of this parent study were made available to us by Genzyme for the conduct of the present study. Favourable changes in LDL-C and apoB100 levels were observed in the parent study. The clearance of plasma LDL-C via the LDL receptor (LDLR) pathway is mitigated by PCSK9 which, by binding to LDLR, prevents the latter from being recycled and hastens their degradation [5].

We hypothesize that PCSK9 may be associated with the observed changes in LDL-C and apoB100 levels. To this end, we compared lipid profile, apolipoproteins, oxLDL, circulating PCSK9 and HDL functionality in five NPD-B patients with a reference group. The latter was made up of age and sex matched healthy individuals. They served to provide reference ranges as a non-disease population for experimental assays and they were used as a comparison group for all measured parameters. We evaluated changes in parameters measured after olipudase alfa. ERT was reported to cause transient elevations of some systemic inflammatory markers [4]. We looked at whether ERT was associated with changes in TNF-α, another marker of inflammation.

## Methods

### Study design

This is an Investigator Initiated Study supported by a research grant from Sanofi-Genzyme for its design and conduct. The study was sponsored by Manchester University NHS Foundation Trust.

The parent study was a phase 1, open-label, within-patient, repeat-dose, dose-escalation study as previously described [4]. In brief, five adult patients (3 males and 2 females) with NPD-B were recruited to the parent study. Informed consent was obtained from all participants before the conduct of any study-related procedures. Participants consented to the storage of samples from the study for use in future ethically approved studies. All 5 patients had hepatosplenomegaly and 4 had thrombocytopenia. 2 of the patients were on stable regimen of lipid-lowering therapy, viz simvastatin 20 mg and 40 mg daily respectively and the statin dose was unchanged during the study. Patients received escalating doses (0.1 to 3.0 mg/kg) of olipudase alfa intravenously. The initial dose of 0.1 mg/kg was given on day 1. This was followed 2 weeks later by 0.3 mg/kg. After 2 consecutive doses of 0.3 mg/kg, dose escalation continued at 0.6, 1.0, 2.0 and 3.0 mg/kg, the last dose was maintained until week 26. Patients who experienced adverse events more than mild in severity either stayed on the same dose or received a reduced dose at the next infusion. All patients were successfully escalated to the target dose of 3.0 mg/kg and they all completed the study at week 26.

Samples made available to us from the parent study included pre-ERT serum samples and plasma samples pre- and post-ERT at 4 different time points: day 1, week 8, week 16 and week 26. All the blood samples at these time points were taken 24 h post-infusion. All study samples were taken in the fasting state. The ERT doses at these times points were 0.1 mg/kg, 1.0 mg/kg, 3.0 mg/kg and 3.0 mg/kg respectively. All samples had been stored at − 80 °C and were transported to our site on dry ice.

For our study, fifteen age- and sex-matched healthy participants were recruited as a reference group from Manchester University NHS Foundation Trust (Manchester, UK) and University of Manchester (Manchester, UK). Reference subjects had no significant pre-existing medical conditions and were not on any regular medications. Patient Information Sheet was given to all eligible subjects and informed consent was obtained from those who agreed to participate before a single fasting blood sample of 60 ml was obtained from each reference subject.

### Laboratory methods

Total cholesterol, triglycerides, direct high-density lipoprotein cholesterol (HDL-C), small dense low-density lipoprotein cholesterol (sdLDL-C), apolipoprotein A1 (apoA1), and apolipoprotein B100 (apoB100) were measured using an RX Daytona auto-analyzer (Randox Laboratories Ltd, Crumlin, UK). Low-density lipoprotein cholesterol (LDL-C) was calculated using the Friedewald equation  [6].

ApoB-depleted serum prepared and cholesterol efflux assay was conducted as described before [7, 8]. Briefly, J774A.1 cells were pelleted and incubated for 24 h with 0.2 µCi of radiolabelled ^3^H-cholesterol in RPMI 1640 medium with 0.2% BSA at 37 °C in a humidified atmosphere containing 5% carbon dioxide. ABCA1 is upregulated using medium containing 0.3 mM C-AMP (8-(4-Chlorophenylthio) adenosine 3′,5′-cyclic monophosphate sodium salt) for 4 h. These cells were then incubated with 2.8% apoB-depleted serum using polyethylene glycol (PEG MW8000) for 4 h. After incubation, the cell media were collected and cells were washed with PBS and dissolved in 0.5 mL 0.2 N NaOH to determine radioactivity. Cellular cholesterol efflux was expressed as the percentage of radioactivity in the medium from the radioactivity in the cells and medium collectively. The intra-assay and inter-assay coefficients of variation were 3.9% and 7.3% respectively. Cholesterol efflux was calculated using the following formula:

$${\text{Cholesterol}}\,{\text{efflux}}\,(\% ) = \frac{{{\text{Radioactivity}}\,{\text{in}}\,{\text{medium}}}}{{{\text{Radioactivity}}\,{\text{in}}\,{\text{cell}}\, + \,{\text{radioactivity}}\,{\text{in}}\,{\text{medium}}}} \times 100$$

Serum paraoxonase (PON-1) activity was determined using paraoxon (O,O-Diethyl O-(4-nitrophenyl) phosphate) as a substrate (Sigma-Aldrich Company Ltd) using a RX Daytona auto-analyzer (Randox Laboratories Ltd) [9]. The intra-assay and inter-assay coefficients of variation were 3.5% and 2.7%, respectively, for the measurement of PON1 activity.

The following tests were done by enzyme linked immunosorbent assay (ELISA) methods: oxLDL [10] and apoB48 [11] (Elabscience, Houston, USA.), intra- and inter-assay coefficients of variation 5.6% and 5.3%, and 4.9% and 7.7% respectively; PCSK9 mass [12] (R&D Systems, Abingdon, UK.), intra-assay coefficient of variation 4.9%; TNF-α [13] (Invitrogen, Fisher Scientific UK Ltd.), intra- and inter-assay coefficients of variation 8.5% and 9.8%.

### Statistical analyses

Statistical analyses were performed using SPSS for Mac (Version 23.0, IBM SPSS Statistics, Armonk, New York, USA) and figures were produced using GraphPad Prism for Mac (Version 7.00, GraphPad Software, La Jolla California, USA). Data were presented as mean and standard deviation for all variables. The independent samples t-test was used for comparison between NPD-B and control groups. A *P* value of less than 0.05 was considered to be statistically significant. Changes after ERT is expressed as percentage.

## Results

The baseline characteristics for NPD-B patients and reference group are presented in Table 1. Compared to reference subjects, NPD-B patients had higher triglycerides, apoB48, oxLDL, PCSK9 mass, TNF-α, and lower HDL-C and apoA1.

**Table 1:** Comparison of lipid profile, markers of LDL quality and HDL functionality, and markers of inflammation between patients with Niemann–Pick disease type B and healthy controls at baseline

	Controls (n = 15)	Niemann–Pick Disease type B (n = 5)	*P* value
Age	35.3 ± 16.3	32.6 ± 9.4	0.363
Total cholesterol (mmol/l)	5.1 ± 1.5	4.5 ± 0.8	0.426
Triglyceride (mmol/l)	1.0 ± 0.3	2.2 ± 1.5	0.003
HDL-C (mmol/l)	1.29 ± 0.27	0.79 ± 0.28	0.002
LDL-C (mmol/l)	3.4 ± 1.3	2.9 ± 0.5	0.469
ApoA1 (g/l)	1.39 ± 0.17	0.84 ± 0.25	< 0.001
ApoB100 (g/l)	0.72 ± 0.26	0.94 ± 0.21	0.098
ApoB48 (µg/ml)	61.0 ± 12.3	89.8 ± 11.9	< 0.001
sdLDL-C (mg/dl)	29.0 ± 18.5	44.7 ± 18.2	0.118
oxLDL (ng/ml)	88.2 ± 21.4	131.1 ± 22.8	0.001
PCSK9 mass (ng/ml)	366.8 ± 127.8	599.0 ± 237.6	0.011
TNF-α (pg/ml)	27.7 ± 8.8	76.0 ± 10.7	< 0.001
PON1 activity (nmol/ml/min)	158.1 ± 128.8	98.5 ± 79.0	0.347
Cholesterol efflux capacity (%)	13.1 ± 1.4	11.9 ± 1.4	0.150

Data are presented as mean ± SD

*P* value for comparison with controls using independent samples t-test

apoA1, apolipoprotein A1; apoB-100, apolipoprotein B-100; apoB-48, apolipoprotein B-48; HDL-C, high-density lipoprotein cholesterol; LDL-C, low-density lipoprotein cholesterol; oxLDL, oxidised low-density lipoprotein; PCSK9, proprotein convertase subtilisin/kexin type 9; PON1, paraoxonase-1; sdLDL-C, small dense low-density lipoprotein cholesterol; TNF-α, tumour necrosis factor α

**Fig. 1:**
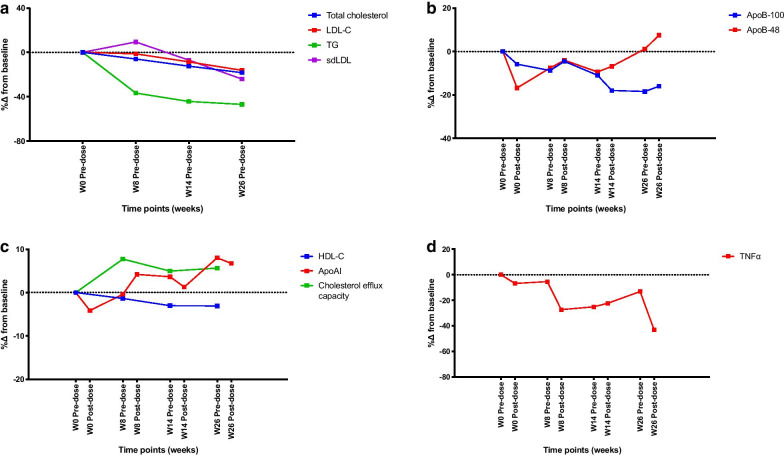
Percentages changes in variables from baseline to week 26. **a** Mean percentage changes in lipid profile from baseline to week 26. **b** Mean percentage changes in apoB-100 and apoB-48 from baseline to week 26. **c** Mean percentage changes in HDL-C, apoAI and cholesterol efflux capacity from baseline to week 26. **d** Mean percentage change in TNFα from baseline to week 26. Absolute values and standard deviations at baseline and week 26 are shown in Table 2. Abbreviations: apoAI, apolipoprotein A-I; apoB-100, apolipoprotein B-100; apoB-48, apolipoprotein B-48; HDL-C, high-density lipoprotein cholesterol; LDL, low-density lipoprotein cholesterol; sdLDL, small dense low-density lipoprotein; TG, triglyceride; TNFα, tumour necrosis factor alpha

**Fig. 2:**
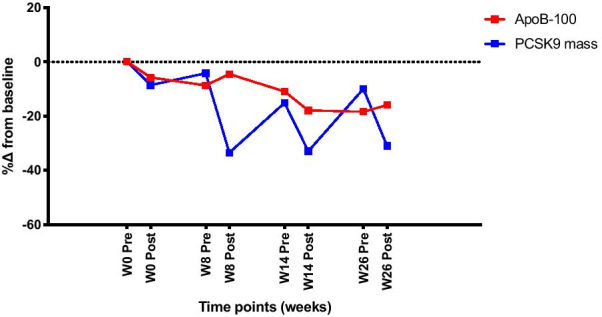
Relationship between percentage change in PCSK9 and ApoB-100 following from baseline to week 26 on olipudase alfa. Absolute values and standard deviations are shown in Table 2. Abbreviations: apoB-100, apolipoprotein B-100; PCSK9, proprotein convertase subtilisin/kexin type 9

Absolute values and changes in measured parameters at baseline and following ERT were shown in Table 2 and Fig. 1 respectively. There were reductions in total cholesterol, triglycerides, LDL-C, apoB100, sdLDL-C, PCSK9 and TNF-α following ERT. PCSK9 level showed consistent reductions after each dosing with olipudase alfa and progressive reductions in apoB100 was observed in conjunction (Fig. 2).

**Table 2:** Lipid profile, markers of lipoprotein quality and functionality, and markers of inflammation of patients with Niemann–Pick disease type B before and after ERT

	Niemann–Pick disease type B (n = 5)			
	Baseline	Week 26	Week 26 change from baseline	Week 26 % change from baseline
Total cholesterol (mmol/l)	4.5 ± 0.8	3.6 ± 0.7	− 0.9 ± 1.0*	− 18.3 ± 20.4*
Triglyceride (mmol/l)	2.2 ± 1.5	1.0 ± 0.4	− 1.2 ± 1.1*	− 46.9 ± 17.3*
HDL-C (mmol/l)	0.79 ± 0.28	0.74 ± 0.24	− 0.04 ± 0.18*	− 3.1 ± 23.3*
LDL-C (mmol/l)	2.9 ± 0.5	2.4 ± 0.5	− 0.5 ± 0.7*	− 16.2 ± 22.1*
ApoA1 (g/l)	0.84 ± 0.25	0.89 ± 0.20	0.05 ± 0.06	8.0 ± 13.3
ApoB100 (g/l)	0.94 ± 0.21	0.75 ± 0.15	− 0.18 ± 0.26	− 18.4 ± 20.4
ApoB48 (µg/ml)	89.8 ± 11.9	92.51 ± 27.3	7.29 ± 12.6	1.2 ± 19.7
sdLDL-C (mg/dl)	44.7 ± 18.2	32.5 ± 13.6	− 12.2 ± 11.2*	− 24.0 ± 29.1*
oxLDL (ng/ml)	131.1 ± 22.8	123.4 ± 37.9	− 14.4 ± 20.8	− 6.7 ± 19.2
PCSK9 mass (ng/ml)	599.0 ± 237.6	492.4 ± 97.6	− 220.8 ± 253.1*	− 10.0 ± 29.1*
TNF-α (pg/ml)	76.0 ± 10.7	65.6 ± 6.5	− 32.7 ± 21.7	− 22.3 ± 7.0

Data are presented as mean ± SD

apoA1, apolipoprotein A1; apoB-100, apolipoprotein B-100; apoB48, apolipoprotein B48; HDL-C, high-density lipoprotein cholesterol; LDL-C, low-density lipoprotein cholesterol; oxLDL, oxidised low-density lipoprotein; PCSK9, proprotein convertase subtilisin/kexin type 9; sdLDL-C, small dense low-density lipoprotein cholesterol; TNF-α, tumour necrosis factor α

^*^Values represent pre-dose measured in serum

## Discussion

In a study of the safety and tolerability of olipudase alfa in patients with NPD-B, an improvement in the total cholesterol, triglyceride and LDL-C was demonstrated [4]. Changes in biomarkers (IL-6, IL-8 and CRP) and hematology variables had already been reported in the original paper [4]. Our study looks at possible associations with changes in lipid parameters and the inflammatory marker TNF-α. Patients with NPD-B have an atherogenic lipid profile characterised by elevated triglyceride, oxLDL, apoB48 and lower HDL-C and apoA1 compared with healthy individuals. The reference group in our study acted as a comparison group not only for experimental assays but for all measured parameters including those that already have standard reference ranges. This is relevant as in the UK, healthy individuals with no history of cardiovascular disease are known to have higher lipid values than standard ranges. Baseline LDL-C level in NPD-B patients was noted to be low. This may be because 2 of the 5 subjects were on stable regimens of lipid lowering therapy [4].It may also reflect hepatic infiltration of sphingomyelin resulting in reduced capacity for VLDL and LDL particle production. We note a discrepancy between our baseline and W26 lipid parameters and those presented in the parent study [4]. We understand the quality of our samples might have been compromised by the processes of freezing and thawing [14] that might have contributed to the discrepancy. Another explanation for the difference in results, particularly noted in HDL-C, might have been the different laboratory assays and analysers used in the parent study and our study.

We observed a prompt reduction in TNF-α following ERT. ERT has been postulated to cause gradual debulking of sphingomyelin releasing ceramide and other sphingomyelin metabolites [4]. TNF-α is known to stimulate sphingomyelinase and trigger ceramide formation, and ceramide in turn is known to negatively regulate TNF-α production [15–17]. One credible explanation of our results could be that as ERT caused ceramide levels to increase, the latter might in turn bring about a reduction in TNF-α. Further studies are needed to confirm this. Another possible reason for the lower TNF-α levels might be the improved wellbeing of ERT recipients as a consequence of lipid accumulation reduction, this would be relevant in the later stages of the study. TNF-α had been shown to induce PCSK9 expression in a cytokine signalling-3 (SOCS3) dependent manner [18, 19] and may therefore be a contributing factor to the reduction in PCSK9 that was also observed following ERT.

ApoB100 is the main apolipoprotein component of LDL, VLDL and intermediate-density lipoprotein (IDL) while apoB48 is an intestinal chylomicron particles specific marker [20–22]. The association between apolipoprotein B and cardiovascular disease has been well established in epidemiological studies [23, 24]. Compared with healthy individuals, patients with NPD-B have higher apoB100 levels indicating increased atherogenic particles and higher fasting apoB48 concentration suggesting a delay in processing and clearance of chylomicron particles. PCSK9 belongs to the proprotein convertase family [25] and its binding to LDL receptors (LDLR) facilitates their degradation and results in increased plasma LDL particle and cholesterol concentrations [26]. It has been suggested that PCSK9 also have an additional direct regulatory role in apolipoprotein B degradation independent of LDLR [27]. PCSK9 has been shown to have correlative relationship with apoB containing lipoproteins including sdLDL and oxLDL [28]. It is possible that the reduction in TNF-α led to lower PCSK9 levels that resulted in better clearance of apoB-containing particles and reductions in sdLDL and oxLDL. The lowering of oxidised LDL particles in turn could lead to further reduced secretion of cytokines.

This study is limited by the small number of subjects with NPD-B receiving ERT with olipudase alfa, limiting the ability to draw strong conclusions from the associations between changes in variables observed. We observed a change in lipid profile and a reduction in TNF-α. We noted that 2 of the 5 subjects in the parent study were on lipid lowering medications and it was not known whether their compliance with medications might have improved during the study as a result of regular contact with healthcare professionals. We analysed samples that had been frozen since the parent study was conducted and it was possible that some sample quality might have suffered. It is important to acknowledge that NPD-B is a very rare condition hence it is not possible to recruit large number of patients.

## Conclusion

We conclude that ERT with olipudase alfa is associated with changes in lipid profile and reduction in a systemic inflammatory marker. Whether this translates to any effects on atherosclerotic cardiovascular risk requires further study.

## Data Availability

The datasets used and/or analysed during the current study are available from the corresponding author on reasonable request.
